# Comprehensive Utilization of Formation Water Scale to Prepare Controllable Size CaCO_3_ Nanoparticles: A New Method to Improve Oil Recovery

**DOI:** 10.3390/nano14171452

**Published:** 2024-09-06

**Authors:** Bo Huang, Shengzhen Hou, Zhao Hua, Jian Zhang, Huan Yang, Yuejun Zhu, Yumiao Tang, Benru Wang

**Affiliations:** 1 State Key Laboratory of Offshore Oil and Gas Exploitation, Beijing 102209, China; huangbo@cnooc.com.cn (B.H.); huazhao@cnooc.com.cn (Z.H.); zhangjian@cnooc.com.cn (J.Z.); zhuyj3@cnooc.com.cn (Y.Z.); 2 CNOOC Research Institute Co., Ltd., Beijing 100028, China; 3 School of Chemistry and Environmental Engineering, Yangtze University, Jingzhou 434020, China; 2022710239@yangtzeu.edu.cn (Y.T.); 2023710233@yangtzeu.edu.cn (B.W.)

**Keywords:** formation water, scale, enhanced oil recovery, calcium carbonate nanoparticles

## Abstract

Formation water scale blocks pipelines and results in oil/gas production decreasing and energy consumption increasing. Many methods have been developed to inhibit scale formation. However, these previous methods are limited by their complications and low efficiency. A new method is proposed in this paper that uses the scale in formation water as a nanomaterial to improve oil recovery via controlling particle size. A series of ligands were synthesized and characterized. Micrometer-CaCO_3_ was formed and accumulated to form scale of a large size under uncontrolled conditions. The tetradentate ligands (L^4^) exhibited an excellent capturing yield of Ca^2+^ (87%). The particle size was very small, but they accumulated to form large particles (approximately 1300 nm) in the presence of Na_2_CO_3_. The size of the CaCO_3_ could be further controlled by poly(aspartic acid) to form sizes of about 700 nm. The flooding test showed that this material effectively improved oil recovery from 55.2% without nano CaCO_3_ to 61.5% with nano CaCO_3_. This paves a new pathway for the utilization of Ca^2+^ in formation water.

## 1. Introduction

With the continuous development of modern society, the demand for oil and gas resources is gradually increasing [1,2,3]. Upon entering the middle and later stages of oilfield maturity, the composition of formation water becomes complicated. High-valent metal ions in formation water, such as Ca^2+^ and Mg^2+^, can form scale on the surface of pipelines and facilities and lead to blockages [4,5,6]. Moreover, these high-valent ions can also react with pipelines, which leads to severe corrosion in water injection systems [7,8,9]. The formation of scale is very complex, involving thermodynamics, dynamics, fluid dynamics, etc. The scale can block the pipelines, resulting in oil and gas production decreasing, energy consumption increasing, unnormal continuous production, or even the stopping of production. In order to inhibit scale formation in oilfields, many methods have been developed, including chemical anti-scaling agents and physical anti-scaling methods [10,11]. However, owing to their complications, the effect of these methods on inhibiting scale formation is limited [12].

In addition, in different oilfields, the types and contents of ions are also different, leading to many uncertain factors and unsatisfactory results when using traditional chemical methods for anti-scaling [12]. Normally, one needs to add much more agent to achieve the anticipated aim. Physical methods, such as ultrasound method for anti-scaling, are as of now immature and easily affected by on-site conditions [13,14]. Most importantly, physical methods must be coupled with chemical methods to treat scale formation. Therefore, although chemical methods are not universally applicable, their low cost, mature technology, fast effectiveness, and good results make them the main means of removing scale [15,16,17,18]. However, chemical agents may remain in formation water after removing scale and gradually accumulate, leading to more serious side effects [19]. Therefore, new methods need to be developed to remove or treat scale.

Owing to their decreasing interfacial tension, changing wetting, enhancing the stability of emulsions, and decreasing injection pressure, many nanomaterials have been extensively utilized in oilfields to enhance oil recovery [20,21,22]. Polyacrylamide (PAM) microsphere and its emulsions have been demonstrated in some oilfields, such as the Changqing Oilfield in China, not only to have excellent dispersion performance, but also to be capable of increasing flow resistance and forcing flow steering to increase swept volume in deep or large channels when the nanospheres flow into the reservoir [23]. Scale formation is a slow process in formation water that involves crystal growth, including homogeneous and heterogeneous crystallization [24]. Therefore, it suggests the probability of controlling the size of scale particles during scale formation in nanometers or micrometers as an inorganic material to enhance oil recovery. This can not only solve the scale problem, but also achieve Ca^2+^ and Mg^2+^ sustainability in formation water [25,26].

**Figure 1:**
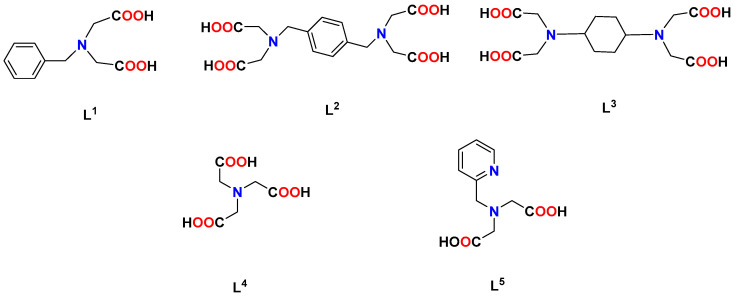
The structures of different ligands.

Normally, CaCO_3_ and MgCO_3_ are precipitated to form scale in formation water owing to their relatively high content of Ca^2+^ and Mg^2+^. To prevent the rapid precipitation of CaCO_3_ and MgCO_3_, the fast capture of the above ions in formation water is a critical step. For instance, chelated agents such as ethylenediamine tetra-acetic acid (EDTA) can be used to capture Ca^2+^ and Mg^2+^ ions and form a very stable complex [27,28], which would be beneficial to the formation of nano- or micrometer CaCO_3_ and MgCO_3_. Moreover, the stability of the chelation should not be too strong, so as to facilitate the fast release metal ions and the in situ formation of nano- or micro-nanoparticles of CaCO_3_ and MgCO_3_ [29,30,31]. Therefore, a suitable ligand is a key factor in the formation of micro-nanoparticles of CaCO_3_ and MgCO_3_. To this end, different kinds of compounds have been designed and synthesized (Figure 1) to capture Ca^2+^ and Mg^2+^. The objectives of this paper include: (1) preparing compounds to capture Ca^2+^ and Mg^2+^; (2) forming micro-nanoparticles of CaCO_3_ and enhancing oil recovery; and (3) elucidating a possible mechanism under present conditions.

## 2. Experimental Section

### 2.1. Materials

Chloroacetic acid (moderate toxicity); sodium hydroxide (NaOH); benzylamine; hydrochloric acid; sodium carbonate; butanediamine; p-xylylenediamine; ammonium chloride (low toxicity); iminodiacetic acid (low toxicity); absolute ethanol; 2-picolyl chloride-HCl (low toxicity); methanol (trace toxicity); poly (aspartic acid) (PASP) (trace toxicity); CaCl_2_; and NaHCO_3_ were purchased from Acros Organic Chemical Company (Waltham, MA, USA). They are analytical grade and without further purification.

### 2.2. Instruments

Fourier-transform infrared spectroscopy (FTIR): VERTEX 70 FT-IR spectrometer Thermo Fisher Technologies (Waltham, MA, USA); ^1^H nuclear magnetic resonance spectroscopy: 400 MHz AVANCE III 600 M (Bruker, Berlin, Germany); X-ray diffraction (XRD): D8 Advance (Dutch Panaco, Almelo, The Netherlands); S3500 laser particle size analyzer(Microtrac, Montgomeryville, PA, USA); Nano ZS Malvern laser particle sizer (Masterizer, Micro, Buckinghamshire, UK); scanning electron microscopy (SEM-EDS): Merlin (Zeiss, Jena, Germany); mass spectrometer: Agilent 6520 Q-TOF LC/MS (Agilent Technologies, Santa Clara, CA, USA); atomic absorption spectrophotometer: AA-7003 (East-west Analytical Instruments Co., Ltd., Beijing, China). 

### 2.3. Preparation of Ion Regulators

N-Benzyl iminodiacetic acid (L^1^): Chloroacetic acid (9.5 g, 100 μmol) in H_2_O (35 mL) was cooled to 50 °C and neutralized with NaOH solution (0.17 g/mL). The alkali was added slowly to avoid the solution temperature exceeding 20 °C. Then, benzylamine (50 μmol, 5.5 mL) was added and the mixture was heated at reflux for one hour. The NaOH solution was added to mixture dropwise, and the mixture was further heated under reflux for one hour. After stopping heating, the mixture’s pH value was adjusted to neutral with hydrochloric acid. The precipitation was formed and collected with a 64% yield.

2,2′,2″,2′′′-((1,4-phenylenebis(methylene))bis(azanetriyl))tetraacetic acid (L^2^): 5.25 g of chloroacetic acid was weighed and dissolved in water. The mixture was stirred and neutralized with sodium carbonate until the pH was 6.8–7. According to the ratio of chloroacetic acid:butanediamine = 6.7:1 (mass ratio), the sodium chloroacetate solution was first transferred into a three-necked flask, then stirred. Next, 906 μL (0.78 g) of butanediamine and 25% NaOH mixture was slowly added to the three-necked flask using a drip funnel. After addition, the pH was adjusted to 9–9.5 using a 25% NaOH solution, and the reaction temperature was maintained at 90–95 °C until the pH value of the solution did not decrease (at the end of the reaction, the pH was maintained at around 9). The reaction solution was cooled down to 40–45 °C, then continuously stirred while 1:1 hydrochloric acid was added for acidification. When the pH of the solution reached 1, no more acid was added and the solution was stirred until precipitation formed. The yield was 64%.

Trans-2,2′,2″,2′′′-(cyclohexane-1,4-diylbis(azanetriyl))tetraacetic acid (L^3^): An aqueous solution of chloroacetic acid (4.75 g; 50 μmol in 17.5 mL) was cooled in an ice bath to 5 °C and neutralized with an aqueous sodium hydroxide solution (2.0 g; 50 μmol in 10 mL). The alkali was added at such a rate that the temperature of the reaction mixture never reached 20 °C. Then, 3.40 g (25 μmol) of p-xylylenediamine was added. The reaction mixture was refluxed and a solution of 2.0 g NaOH in 10 mL of water was added dropwise to it over one hour. A clear colorless solution was formed, and the refluxing was continued for one additional hour. The reaction mixture was poured into a large beaker, acidified to pH = 2 with diluted hydrochloric acid (1:1 *v*/*v*), and cooled to room temperature. The bottom of the beaker was scratched with a sharp glass rod to induce precipitation and stirred until the mixture solidified. After standing overnight in a refrigerator, the product was filtered off by suction, washed with ice water, and dried. The yield was 57%.

Nitrilotriacetic acid (L^4^): 8.85 g monochloroacetic acid was put into the flask, stirred and heated until molten, and then an aqueous solution of NaOH was added (3.70 g in 10 mL). The solution was maintained at 80–90 °C for 5–6 h. Then, it was concentrated under vacuum, heated to 60 °C, and adjusted to a pH of 7–8 with saturated sodium carbonate solution. Under 80 °C, 4.0 g saturated solution of ammonium chloride was slowly added. Then the pH was adjusted to 9–10 with 1 M NaOH. After standing for 4 h at the room temperature, the mother liquors were then acidified (pH = 1) by the addition of HCl (6 M) and the product was removed by filtration, washed (cool water) and air-dried. The yield was 66%.

[Carboxymethyl(pyridin-2-ylmethyl)amino]acetic acid (L^5^): Iminodiacetic acid (6.55 g, 50 mmol) was added to a solution of 4.0 g (100 mmol) of NaOH in 20 mL of H_2_O and 60 mL of absolute ethanol. The solution was stirred vigorously. Solutions of 8.2 g (50 mmol) of 2-picolyl chloride-HCl in 17 mL of H_2_O and 4.0 g (100 mmol) of NaOH in 7 mL of H_2_O in separate addition funnels were then added over a period of 8 min. The reaction mixture was warmed to 70 °C and left to stir for 4 h. Then, an additional 4.0 g of NaOH was added, and the reaction mixture was stirred for another hour, after which the amber solution was evaporated, leaving a yellow solid. 50 mL of H_2_O was added, and the solution was acidified with concentrated HCl to pH 1.5. The white solid obtained was recrystallized from methanol. The yield was 35.7%.

The five ligands were characterized by FTIR and ^1^HNMR. The samples were characterized by FTIR using the potassium bromide compression method with a scanning range of 4000–500 cm^−1^ and 32 scans. The solvent choice for ^1^H NMR characterization was water.

### 2.4. Properties of Scale under Uncontrolled Condition

In order to explore the effect of ligands on the size and morphology of coalesced particles, the coalesced particles without ligand control were first analyzed. After the simulated formation water had sat for 30 d, the scale in the water was filtered, dried in an oven, and then characterized by powder X-ray diffraction (XRD). The test conditions were as follows: operating voltage, 40 kV; operating current, 40 mA; scanning range, 5° to 40°; scanning speed, 2°/min. Scale particle size was analyzed with a S3500 laser particle size analyzer. The simulated formation water scale was treated ultrasonically for 20 min to test the particle size. The surface morphology of the particles was characterized by field emission scanning electron microscopy (SEM). A small amount of the sample was taken and pasted on a conductive adhesive and tested after gold spraying. The composition of the scale samples was determined by energy dispersive spectrometer (EDS). The sample was placed in an SEM sample chamber equipped with an EDS detector, which was used to analyze the chemical composition of the sample by converting its X-ray signal into energy spectrum data.

### 2.5. The Capturing of Ca^2+^ by L^1^–L^5^

In order to study the reaction of these ligands with Ca^2+^, ESI/MS was used to characterize them. The atomizer flow rate was 3 L/min, the drying gas flow rate was 10 L/min, and the heater flow rate was 10 L/min.

### 2.6. CaCO_3_ Particle Formation by Na_2_CO_3_ or NaHCO_3_

There is a dynamic equilibrium between ligands, CaCl_2_, and Na_2_CO_3_. This section mainly studies the reaction mechanism of ligands and Na_2_CO_3_ to generate CaCO_3_ particles. The stability constant is calculated as follows:

(1) $$K=\frac{\left(AXn\right)c}{\left(A\right)c*\left(X\right)c^{n}}$$

where (*AXn*)*c* represents the concentration of the complex, (*A*)*c* represents the concentration of the core substance, (*X*)*c* represents the concentration of the ligand, and *n* represents the number of ligands.

To determine the presence of micro-nanoparticles, excess Na_2_CO_3_ solution was gradually dripped in and kept at room temperature for at least 4 d to produce a white precipitate. The CaCO_3_ particles under the control of the ligands were characterized by XRD and SEM. The test method was the same as in Section 2.4.

### 2.7. CaCO_3_ Nanoparticles Formation and Capturing Yield via L^1^–L^5^

In order to analyze the effects of L^1^–L^5^ on Ca^2+^ ion capture rate and CaCO_3_ particle size in the presence of NaHCO_3_ solution, the different ligands (L) were added, n_Ca2+_/n_L_ was changed, and the concentration of free Ca^2+^ ions was determined by atomic absorption spectrometry after sitting for 72 h. Plasma gas flow was 12 L/min, nebulizer flow was 0.7 L/min, and auxiliary gas flow was 0.4 L/min. After 20 min of sonication, the was placed in a cuvette and the particle size of the CaCO_3_ produced by the different ligands at different concentrations was measured using a Malvern laser particle size analyzer.

It was found that the ratio of n_Ca2+_/n_NaHCO3_ also had an effect on the formation of CaCO_3_, but there was no obvious rule among the different ligands. In this paper, the L^4^ ligand was selected to set different proportions of n_Ca2+_/n_NaHCO3_. The concentration of free Ca^2+^ ions and the particle size of the CaCO_3_ produced under different ratios of n_Ca2+_/n_NaHCO3_ were determined by atomic absorption spectrometry and Malvern laser particle size analyzer, respectively, to determine the best ratio of n_Ca2+_/n_NaHCO3_.

Since the particle size of CaCO_3_ particles is still large, it is necessary to further adjust the particle size by adding appropriate chemical reagents. According to previous studies, charged polymers such as polyaspartate (PASP) [31] and polyacrylic acid (PAA) [32] can stabilize amorphous calcium carbonate and may have mineralizing activity. In this paper, PASP was used to further control the size of CaCO_3_ particles. The amount of added polyaspartic acid (PASP) was changed, and the particle size under different PASP dosages was tested by Marvin laser particle size analyzer to explore the effect of the addition amount on the particle size of CaCO_3_.

### 2.8. The Flooding Experiment

**Figure 2:**
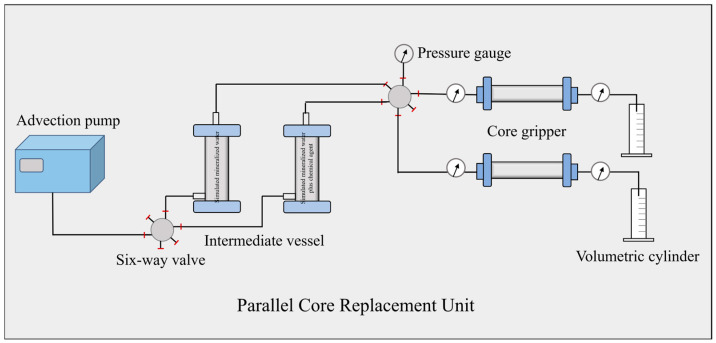
The core displacement device.

A single core replacement experiment was used to simulate the oil flooding of agglomerated particles. The water used in the experiment was simulated formation water, with a salinity of 107,216 mg/L; its main components and concentration are listed in the Table 1. The simulated formation water refers to the formation water of an offshore oil field in China, which is highly mineralised and can reflect the effect of an agent well. The oil used in the experiment was simulated oil, which was obtained by mixing crude oil and aviation paraffin in a ratio of 1:1 (density: 0.8893 g/cm^3^, viscosity: 17.5 mPa·s (50 °C)). An artificially homogeneous cylindrical core with a size of Ø2.5 × 10 cm was used for the experiment. The detailed experimental procedure was as follows:

1. Firstly, simulated formation water was placed in an intermediate container and injected into the core at a flow rate of 1 mL/min to establish the original water saturation. Core dry weight m_1_ and core wet weight m_2_ after water injection were recorded, respectively.
2. Simulated oil was then injected into the core to establish the original oil saturation. The simulated formation water was then injected into the core at a flow rate of 1 mL/min until no oil was produced. The injection volume and differential pressure were recorded, respectively, and the permeability was calculated via Darcy’s equation to get the recovery rate of the water flooding.
3. The simulated formation water with and without additive were placed in an intermediate container, respectively. The water drive recovery rate was calculated by injecting the core at a flow rate of 1 mL/min when the pressure was stable and no oil was produced. The experiment was repeated for each solution to avoid error. The detailed information can be seen in Figure 2.

The relevant calculation formula is as follows:

(2) $$V=\frac{m_{2}-m_{1}}{\rho }$$

where *V* represents the effective pore volume in mL, *m*_1_ represents the dry weight of the core in kg, *m*_2_ represents the wet weight of the core in kg, and *ρ* represents the simulated formation water density in g/cm^3^.

(3) $$n=\frac{V}{V_{1}}$$

where *n* represents effective porosity, dimensionless; *V* represents effective pore volume in mL; and *V*_1_ represents core volume in mL.

(4) $$K=\frac{Q\mu l}{∆PA}$$

where *K* represents the permeability of the aqueous phase in units of 10^−3^ μm^2^; *Q* represents the fluid flow rate through the core per unit time in units of cm^3^/s; *µ* represents the viscosity of the fluid in units of mPa·s; *L* represents the length of the core in units of cm; Δ*P* represents the difference in pressure between the inlet and outlet in units of MPa; and *A* represents the cross-sectional area of the core in units of cm^2^.

## 3. Results and Discussion

### 3.1. Preparation of Ion Regulator

N-Benzyl iminodiacetic acid (L^1^): The elemental analysis of calculation result for C_11_H_13_NO_4_ was C:H:N = 59.19%:5.87%:6.27% and the test results were C:H:N = 59.17%:5.89%:6.28%. The typical chemical shifts were δ 2.94 (s, 4H), 3.59 (s, 2H) and 7.26 (m, 5H).

**Figure 3:**
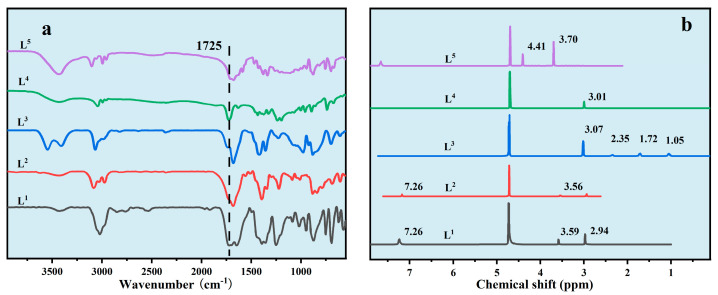
Spectra of compounds, (**a**): FTIR results of ligands; (**b**): ^1^H NMR results of ligands.

2,2′,2″,2′′′-((1,4-phenylenebis(methylene))bis(azanetriyl))tetraacetic acid (L^2^): Figure 3 shows the FTIR result as υ(C=O) in 1732 and 1720 cm^−1^. The elemental analysis of calculation result for C_16_H_20_N_2_O_8_ was C:H:N = 52.17%:5.47%:7.61% and the test results were C:H:N = 52.15%:5.49%:7.64. The typical chemical shifts were δ 3.56 (s, 4H) and 7.26 (s, 4H).

Trans-2,2′,2″,2′′′-(cyclohexane-1,4-diylbis(azanetriyl))tetraacetic acid (L^3^): Figure 3 showed the FTIR result as υ(C=O) 1729 and 1714 cm^−1^. The elemental analysis of calculation result for C_11_H_13_NO_4_ was C:H:N = 48.55%:6.40%:8.09% and the test results were C:H:N = 48.47%: 6.35%: 8.14%. The typical chemical shifts were δ 1.05 (q, 4H), 1.72 (q, 4H), 2.35 (s, 2H), 3.07 (s, 8H).

Nitrilotriacetic acid (L^4^): Figure 3 shows the FTIR result as υ(C=O) 1740 and 1725 cm^−1^. The elemental analysis of calculation result for C_6_H_9_NO_6_ was C:H:N = 37.70%:4.75%:7.33% and the test results were C:H:N = 37.72%:4.71%:7.36%. The typical chemical shift was δ 3.01 (s, 6H).

[Carboxymethyl(pyridin-2-ylmethyl)amino]acetic acid (L^5^): Figure 3 shows the FTIR as υ(C=O) 1727 and 1718 cm^−1^. The elemental analysis of calculation result for C_10_H_12_N_2_O_4_ was C:H:N = 53.57%:5.39%:12.49% and the test results were C:H:N = 53.58%:5.37%:12.51%. The typical chemical shifts were δ 3.70 (s, 4 H), 4.41 (s, 2 H).

### 3.2. Properties of Scale under Uncontrolled Condition

**Figure 4:**
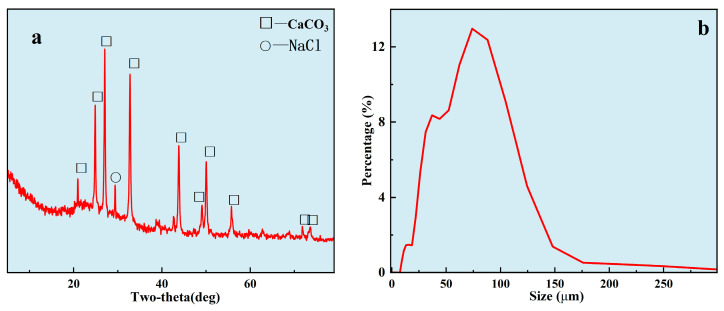
The properties of uncontrolled scale. (**a**): XRD of Simulated scale; (**b**): scale size analysis.

**Figure 5:**
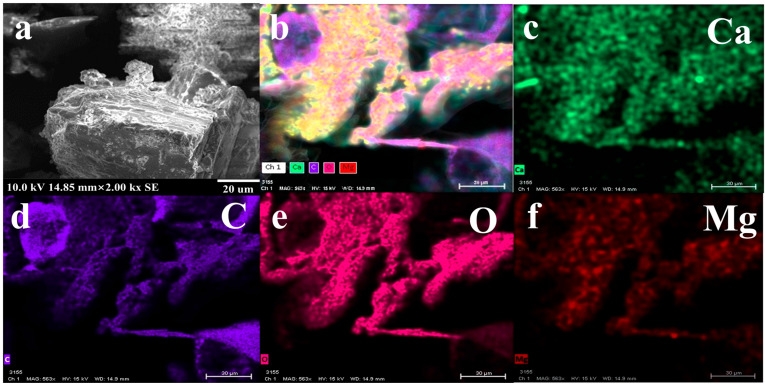
The SEM-EDS of particles, (**a**): SEM result; (**b**): EDS result; (**c**–**f**): C, Ca, O and Mg elements.

First, the scale samples without ligand control were analyzed, as shown in Figure 4. This simulated real scale formation, and especially the size distribution. The XRD of the scale was consistent with the standard diffraction pattern of CaCO_3_ (Figure 4a). The typical peaks were 21.00°, 24. 90°, 27.05°, 32.78°, 43.85°, 50.07°, 55.80°, 71.97°, and 73.59°. It was a typical calcite. The particle size of the scale was analyzed by S3500 laser particle size analyzer (Figure 4b). The median particle diameter (D50) was 56.02 μm. The mean volume diameter, mean number diameter, and mean area diameter were 78.08 μm, 26.64 μm, and 59.02 μm, respectively. It had a high particles size under the uncontrolled condition. The surface morphology of the particles was also characterized by SEM in Figure 5a. The surface of the particles was very rough and irregular under the uncontrolled condition. This indicated that the rough surface improved the specific surface of the particles, resulting in the micro-particles easily adsorbing onto the surface particles to form scale. In other words, it was also necessary to modify the surface of the particles to inhibit the adsorbing of the particles. Furthermore, the surface of composition was initially determined by EDS (Figure 5b–f). This revealed that it was mainly composed of O (38.3%), Ca (36.7%), C (14.3%), and Mg (8.2%). Combined with the results of EDS, the scale was mainly composed of CaCO_3_ (~95.3%). Combining this with Figure 4a,b, it can be confirmed that, under normal conditions, the CaCO_3_ precipitate formed easily and its particle size was big, which means it can block the pipeline easily and cannot be utilized directly. Hence, it is necessary to control the size of the particles and modify the surface of the particles such that they can flow with the formation water, thus realizing the sustainable utilization of Ca^2+^ in formation water.

### 3.3. The Capturing of Ca^2+^ by L^1^–L^5^

**Figure 6:**
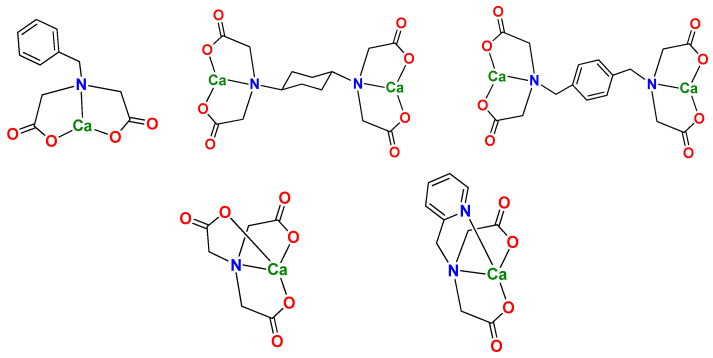
The possible coordination modes for L^1^–L^5^ with Ca^2+^ in H_2_O.

**Figure A1:**
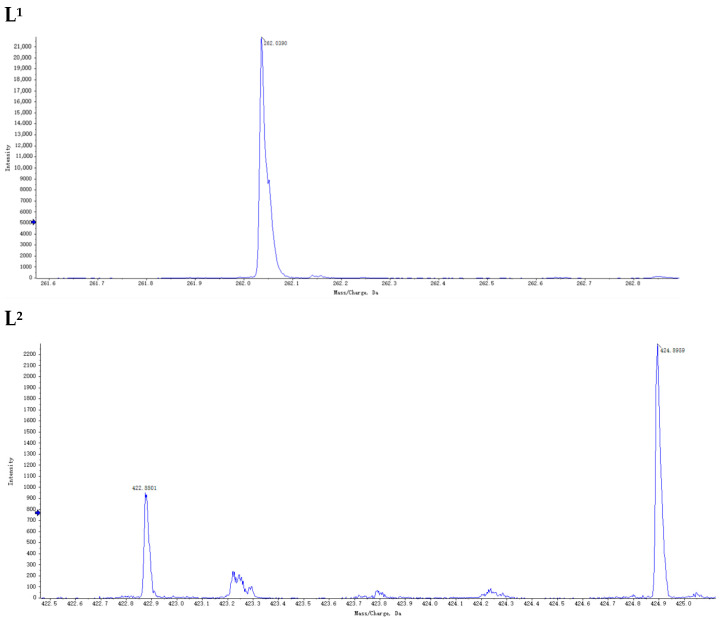

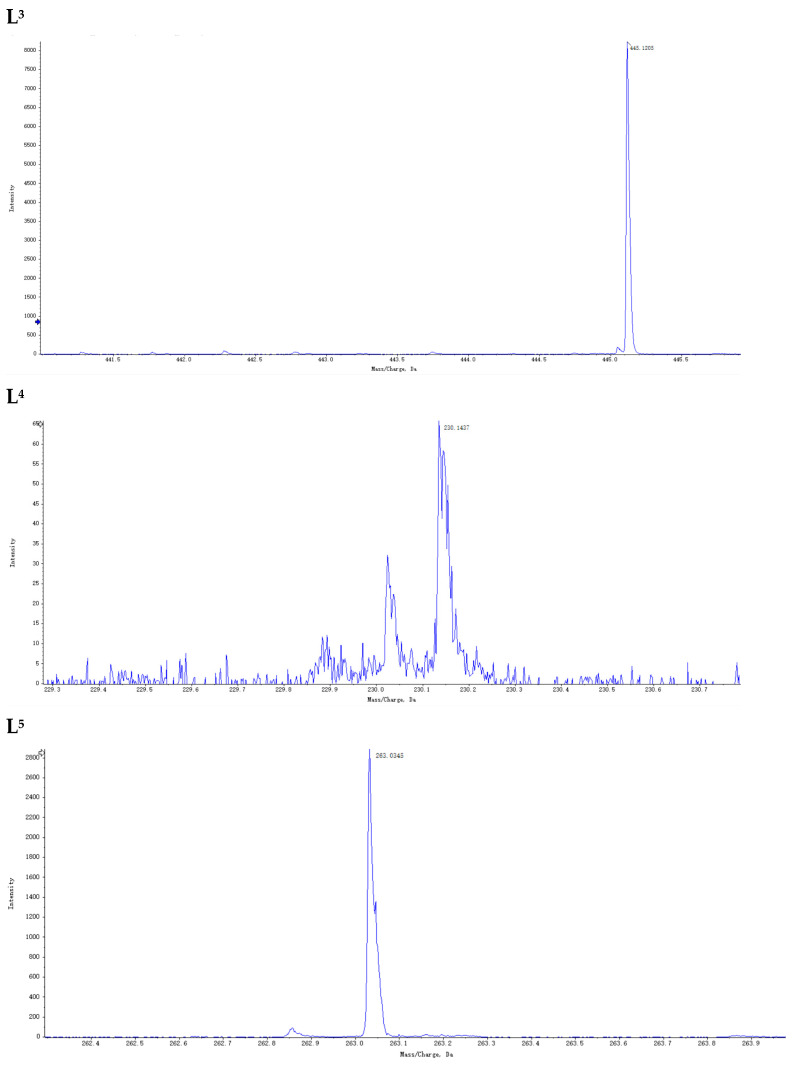
MS results of complexes of Ca^2+^.

All multi-dentate O-containing chelated ligands in this paper could form stable complexes with Ca^2+^ and Mg^2+^. Therein, the reactions of these ligands with Ca^2+^ were investigated. L^1^ was a typical tri-dentate ligand, which could easily chelate Ca^2+^ in H_2_O. The ESI/MS results (Figure A1) showed a predominant peak appearing at *m*/*z* = 262, which was assigned to the species of [L^1^ + Ca^2+^ − H]^+^ (Figure 6). The result indicated that the mole ratio of L^1^ and Ca^2+^ was 1:1. L^2^ and L^3^ were also used to react with Ca^2+^. ESI/MS results showed two peaks appearing at the *m*/*z* = 423, and 445, respectively, which were assigned to [L^2^ + 2Ca^2+^ − 3H]^+^ and [L^3^ + 2Ca^2+^ − 3H]^+^. The mononuclear species were not found due to the steric effect. To tune the solubility in H_2_O, the different cyclohexyl and phenyl groups in L^2^ and L^3^ were used. L^4^ and L^5^ were typical of tetra-dentate ligands and had a stronger ‘chelated effect’ [25] than those of L^1^–L^3^. ESI/MS results showed the predominant peaks appearing at *m*/*z* = 230 and 263, respectively, which were assigned to [L^4^ + Ca^2+^ − H]^+^ and [L^4^ + Ca^2+^ − H]^+^ (Figure 6). From the above analysis, it can be confirmed that Ca^2+^ can be effectively captured by L^1^–L^5^ because more carboxyl functional groups can chelate more Ca^2+^.

### 3.4. CaCO_3_ Particle Formation by Na_2_CO_3_ or NaHCO_3_

Based on the ESI/MS investigation, these additives can quickly form stable complexes with Ca^2+^ when the mole ratio of ligand and Ca^2+^ is 1. The addition of excess Na_2_CO_3_ did not generate any obvious white precipitation, even when the mixture was left standing for over 3 days, with the presence of the previously described complexes, while the white CaCO_3_ precipitation was immediately observed in the absence of these ligands. Although the white CaCO_3_ precipitation could not be observed in the presence of these ligands, micro-nanoparticles were gradually formed. This result revealed the presence of a dynamic equilibrium between the ligand, CaCl_2_, and Na_2_CO_3_. Taking L^1^ as an example, L^1^ can form a complex with Ca^2+^ with the stability constant K_1_ (~1 × 10^9^) (a). The large stability constant indicated that the Ca^2+^ in aqueous phase was nearly completely captured by L^1^. When Na_2_CO_3_ was added, Ca^2+^ would combine with CO_3_^2−^ to generate the micro-nanoparticle of CaCO_3_ (K_sp_ ~ 2.8 × 10^−9^) (b). Thus, the total reaction was (a) + (b) → (c). According to K_1_ × K_2_ = 1/K_sp_, K_2_ = 1/(K_sp_ × K_1_) = 0.36. On the other hand, the formation rate (R) of CaCO_3_ was significantly lowered due to the formation of a stable complex [L·Ca^2+^]. The CaCO_3_ precipitation was not formed in the presence of L^1^ mainly due to the very low concentration of Ca^2+^ in the reaction system.


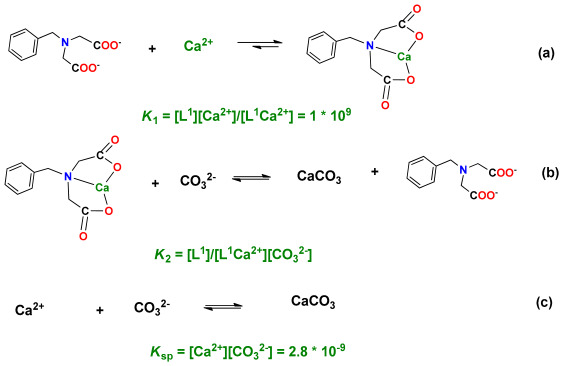


**Figure 7:**
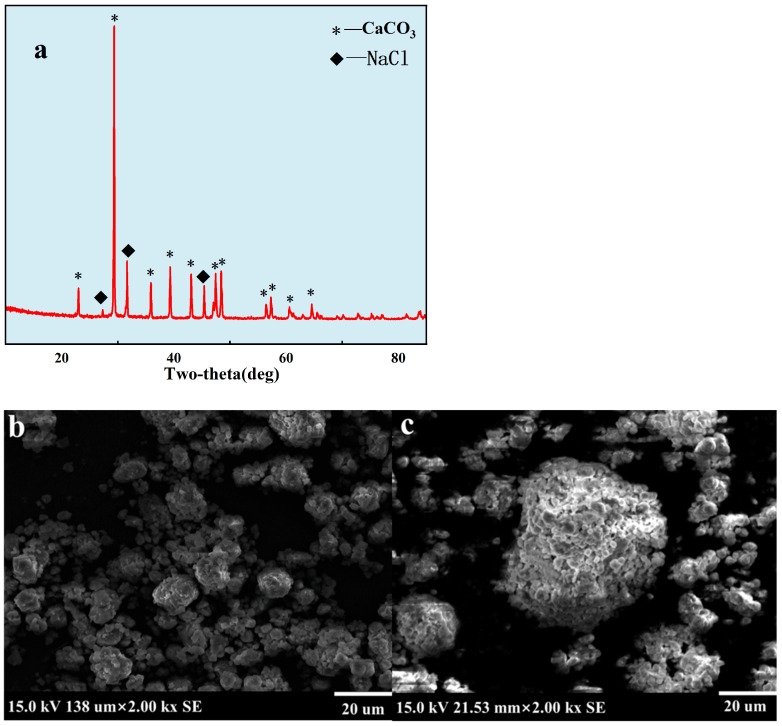
XRD and SEM for CaCO_3_ particles. (**a**): XRD of nano-CaCO_3_ under ligand control; (**b**,**c**): SEM of nano-CaCO_3_ under ligand control.

The XRD pattern was very consistent with that of the standard CaCO_3_ sample. The typical peaks were 23°, 29°, 36°, 39°, 43°, 47.5°, and 48.5°. It was a typical calcite characteristic peak (Figure 7a). SEM showed that a spherical shape could be clearly observed (Figure 7b,c). It also can be observed that the particles were relatively dispersed and showed a size of about 800 nm (Figure 7c), which was a smaller size compared to that of the uncontrolled CaCO_3_ particles. Generally, high specific surface energy with more micro-nanoparticles coalescence to form micrometer-sized particles, and more smaller nanoparticles are expected to be formed under controlled conditions. This provided the possibility of enhanced oil recovery with micro-nanomaterials.

The above experiments demonstrated that synthetic ligands could capture Ca^2+^ effectively in the aqueous phase when mole ratio of L and Ca^2+^ exceeded 1. Thus, when Na_2_CO_3_ was added, the CaCO_3_ precipitate was not formed immediately. However, CaCO_3_ precipitate rapidly formed when mole ratio of ligand and Ca^2+^ was lower than 1. To simulate the real situation in the oilfield, NaHCO_3_ was used instead of Na_2_CO_3_ due to HCO_3_^−^ needing to dissociate to CO_3_^2−^, resulting in a CaCO_3_ precipitate that was relatively slow. Three possible equilibration reactions of NaHCO_3_ occurred.

HCO_3_^−^ + H_2_O = H_2_CO_3_ + OH^−^ (5)

HCO_3_^−^ + H_2_O = CO_3_^2−^ + H_3_O^+^ (6)

H_3_O^+^ + OH^−^ = 2H_2_O (7)

In a typical 0.1 M NaHCO_3_ aqueous solution, the pH value was close to 8.34. c(HCO_3_^−^), c(CO_3_^2−^) and c(H_2_CO_3_) was close to 9.8 × 10^−2^ M, 9.8 × 10^−4^ M, and 9.8 × 10^−4^ M, respectively, (K_a1_(H_2_CO_3_) = 4.5 × 10^−7^ and K_a2_(H_2_CO_3_) = 4.7 × 10^−11^). Thus, c(CO_3_^2−^) was much lower than that of Na_2_CO_3_ and no obvious precipitate was found when NaHCO_3_ was used instead of Na_2_CO_3_ in CaCl_2_ solution.

### 3.5. CaCO_3_ Nanoparticles Formation and Capturing Yield via L^1^–L^5^

The initial concentration of c(Ca^2+^) was 460 mg/L. From Figure 6, it can be found that L^2^ and L^3^ could capture more Ca^2+^, but the capturing yield was not higher in Table 2. Complexes formed with ligands stronger than L^4^ are likely to release less Ca^2+^. The Ca^2+^ in solution can be fixed via two possible pathways: One was the formation of CaCO_3_, and another was the formation of stable complexes with ligands. As shown in Table 2, among the six ligands (L^1^ to L^5^), L^4^ exhibits the max Ca^2+^ capturing yield. When c(L^4^) increased from 125 mg/L to 250 mg/L (n_Ca2+_/n_L4_ 16.5:1 to 8.22:1), the free c(Ca^2+^) in aqueous solution was significantly reduced, and the capturing rate increased from 73.0% to 87%. While the c(L^4^) further increased from 250 mg/L [(n_Ca2+_/n_L4_~8:1)] to 2000 mg/L [(n_Ca2+_/n_L4_~1:1)], the Ca^2+^ capturing yield did not increase significantly. Therefore, the optimal ratio of n_Ca2+_/n_L4_ was ~8:1. The mean area diameter of particle size was near 1300 nm using L^4^. It was lowest among the ligands. In other words, when L^4^ was used, it had highest capturing yield and smallest particle size.

L^1^ was a tridentate ligand and its complex with Ca^2+^ had relatively smaller stability constants than those of L^4^ and L^5^. As shown in Table 2, the capturing yield of Ca^2+^ for L^1^ was in the range of 64.1–69.0%, which was essentially identical to those of the bis(tridentate) ligands L^2^ and L^3^ (61.4–69.0%). The particle sizes of CaCO_3_ for L^1^ (1451 nm~1896 nm) were also similar to those for L^2^–L^3^ (1380–1843 nm). These results indicated that no obvious ‘cooperation effect’ for these bis(tridentate) ligands existed. Actually, the tridentate units of L^1^–L^3^ at the same mass concentration were very close. These ligands, in theory, have similar stability constants. Based on the above results, Ca^2+^ in solutions was mainly bound via the formation of CaCO_3_ rather than Ca^2+^ complexes. 

**Figure 8:**
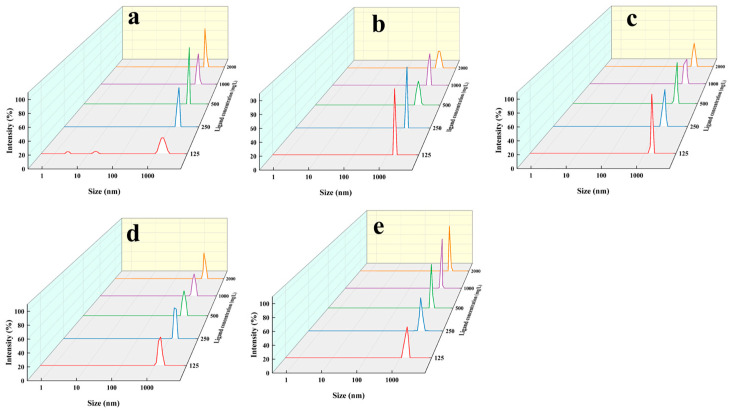
Particle size distribution plots, (**a**): different concentrations of L^1^; (**b**): different concentrations of L^2^; (**c**): different concentrations of L^3^; (**d**): different concentrations of L^4^; (**e**): different concentrations of L^5^.

Among these ligands, L^4^ and L^5^ are tetradentate ligands. As compared with L^1^, L^5^ has an additional pyridyl ring. The capturing yield of L^5^ (65.4–69.0%) was slightly increased as compared with those of L^1^/L^3^. The particle sizes of CaCO_3_ for L^5^ (1469 nm to 1654 nm) were also smaller than those of L^1^ and L^3^. These results revealed that tetra-dentate ligand (L^5^) exhibited a better performance than those of the tridentate ligands (L^1^–L^3^). In L^4^, a carboxyl group was used instead of the pyridyl ring in L. The capturing yield of L^4^ (73.0–87.0%) were higher than that of L^5^. However, the capturing yield of Ca^2+^ did not increase with the increase of the stability constants of the ligands. As the Ca^2+^ needed to be captured and released in a dynamic equilibrium, the suitable stability constant of the ligands to Ca^2+^ was one of the important factors. It was also noteworthy that a complex with an extremely large stability constant was not beneficial to the formation of CaCO_3_ nanoparticles. It was also noted that the particle sizes of CaCO_3_ for L^4^ (1362 to 1467 nm) were the smallest among these ligands. Thus, L^4^ exhibited the best performance on the ion capturing yield and particle size. Although the size of the nanoparticles may be influenced by many factors including the temperature, stirring rate, liquid flow rate, solubility, etc., the stability of the complex that controls the concentration of Ca^2+^ released from the complex was one of the most important factors that then further controlled the formation rate and size of the CaCO_3_ particles (Figure 8). 

Different ratios of n_Ca2+_/n_NaHCO3_ were also investigated. Among these, L^4^ has been selected to investigate the best ratio of n_Ca2+_/n_NaHCO3_ as it showed a max capturing yield when n_Ca2+_/n_L4_ was 8:1. As shown in Table 3, it was clearly observed that the max capturing yield was 87.0% when the n_Ca2+_/n_NaHCO3_ ratio was 0.363 under same conditions.

In Figure 7, it can be found that smaller particles accumulated to form large particles. L^4^ was chosen to demonstrate this. As shown in Table 4, the experiments were conducted with different concentrations of PASP under the optimal ratio (n_Ca2+_/n_L4_ 8:1, n_Ca2+_/n_NaHCO3_ 0.363). The results indicated that the PASP mainly affected the particle size of CaCO_3_, while the capturing yield of Ca^2+^ remained almost unchanged. The particle size of CaCO_3_ in the system without PASP addition was in the range of 1266–1467 nm, while after addition of PASP, the particle size of CaCO_3_ decreased to the range of 764.8–1011 nm. The results showed that the optimal amount of PASP was 500 mg/L in the present system. The results also indicated that PASP probably could react with CaCO_3_. As we all know, hydroxyl can be found on the surface of inorganic matter surfaces. Aspartic acid contains two carboxyl functional groups, which can react with the hydroxyl group on the surface of the CaCO_3_ particle to form more hydrogen bonds. It would inhibit the particles accumulating to form large particles, leading to small particle formation. A high content of additives could form more hydrogen bonds and show relatively small particles of CaCO_3_.

### 3.6. Controllable CaCO_3_ Particles Improved Oil Recovery

**Figure 9:**
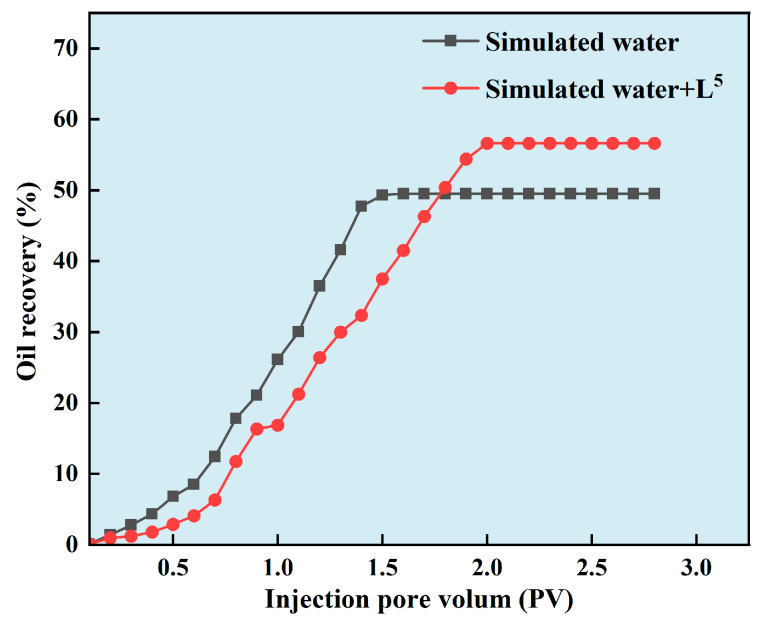
Plot of injection pore volume versus oil recovery.

To investigate the role of particles to enchained oil recovery (EOR), L^4^ was selected for a simulated flooding experiment. As shown in Table 5, oil recovery reached 61.5% when the simulated formation water with L^4^ and PASP was injected, while it was 55.2% when the simulated formation water without additive was injected. In other words, L^4^ was an effective additive to improve EOR. It also demonstrated that the sustainability of Ca^2+^ in formation water was feasible. The results could be attributed to the interaction between L^4^ and the Ca^2+^ present in the formation water, resulting in the micro- or nano-sized agglomerated particles formation during presence of PASP. Once these particles entered the reservoir, they would flow and deposit in the pores, effectively pushing oil droplets forward to improve EOR. On the other hand, the surface of nano CaCO_3_ has more active functional groups like -OH, which would react with oil via hydrogen bond to change the interfacial tension [33,34]. It would benefit to improve EOR. Normally, it has a relatively high interfacial tension under high salinity condition. Hence, it needs to decrease the interfacial tension to improve EOR in formation water due to its high salinity. Normally, the rock surface properties would change from hydrophilic to lipophilic after long-term immersion in crude oil. It needed to change the wettability to enhance the oil film on the rock surface washing away. Tian et al. found that nanofluid changed the contact angle of oil film to improve EOR [34]. Therefore, micro- and nano-sized CaCO_3_ particles had a excellent performance to improve EOR (Figure 9).

**Figure 10:**
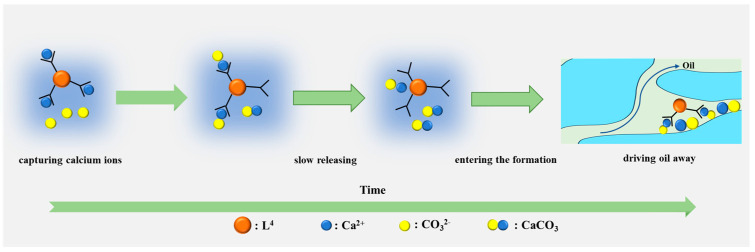
L^4^ displacement mechanism diagram.

The key process in this paper was controlled micro- and nano-sized CaCO_3_ particle formation. Then, when the particles entered the reservoir pores, they exhibited an affinity for oil droplets, which facilitated the displacement of oil droplets within the reservoir. In addition, the presence of these particles could improve pore structure and reduce oil entrapment (Figure 10). Therefore, the efficiency of oil recovery was improved.

## 4. Conclusions

The paper reported the resource utilization of Ca^2+^ in formation water. The resource utilization of Ca^2+^ in formation water was investigated by organic ligands. The synthetic multi-dentate ligands can effectively capture Ca^2+^ in formation water. Then, Ca^2+^ slowly released to form CaCO_3_ nanoparticles. Among the six ligands (L^1^ to L^5^), L^4^ had highest capturing yield and smallest particle size. And it was clearly observed that the max capturing yield was 87.0% when n_Ca2+_/n_NaHCO3_ ration was 0.363 under same conditions. The particle size can be further controlled by PASP. Therefore, adding the ligands studied in this paper to highly mineralized formation water can make calcium ions form calcium carbonate particles of controllable size, thus improving the recovery rate. The flooding test demonstrated it was an effective method, improving the EOR from 55.2% to 61.5%. The results in this study gives a new pathway to utilize the Ca^2+^ in formation water and provides a new method for scale treatment in petrochemical industry.

## Figures and Tables

**Table 1:** The composition of simulated formation water (mg/L).

Na^+^	K^+^	Ca^2+^	Mg^2+^	Cl	SO_4_^2^	HCO_3_^−^	Total Mineralization
11,977	133	695	269	21,939	15	370	35,397

**Table 2:** Ca^2+^ capturing yield and particle size by adding different additives.

Ligands	c(L) (mg/L)	n_Ca2+_/n_L_	c(Ca^2+^) (mg/L)	Capturing Yield (%)	Particle Size (nm)
L^1^	125	38.4:1	150	67.4	1652
	250	19.2:1	144	68.7	1896
	500	9.6:1	142	69.0	1841
	1000	4.8:1	158	65.6	1653
	2000	2.4:1	165	64.1	1451
L^2^	125	31.8:1	152	67.0	1668
	250	15.9:1	150	67.4	1539
	500	7.95:1	143	68.9	1519
	1000	3.98:1	163	64.5	1698
	2000	1.99:1	177	61.4	1843
L^3^	125	60:1	163	64.5	1651
	250	30:1	161	65.0	1614
	500	15:1	157	65.8	1756
	1000	7.5:1	156	66.0	1687
	2000	3.74:1	165	64.1	1563
L^4^	125	16.5:1	124	73.0	1362
	250	8.22:1	60	87.0	1467
	500	4.12:1	80	82.6	1324
	1000	2.06:1	74	83.8	1266
	2000	1.03:1	80	82.5	1385
L^5^	125	38.7:1	157	65.8	1652
	250	19.4:1	150	67.4	1632
	500	9.7:1	143	69.0	1534
	1000	4.84:1	155	66.2	1654
	2000	2.42:1	160	65.4	1469

**Table 3:** Ca^2+^ capturing yield of L^4^ by using different n_Ca2+_/n_NaHCO3_ rations.

Entry	n_Ca2+_/n_L4_	n_Ca2+_/n_NaHCO3_	Capturing Yield (%)
1	8:1	0.726:1	72.3
2	8:1	0.363:1	87.0
3	8:1	0.181:1	81.2
4	8:1	0.09:1	78.4
5	8:1	0.045:1	61.2

**Table 4:** Effect of addition of PASP on Ca^2+^ capturing yield and particle size.

c(L^4^) mg/L	c(PASP) mg/L	n_Ca2+_/n	n_Ca2+_/n_NaHCO3_	Capturing Yield (%)	Particle Size (nm) without PASP	Particle Size (nm) with (PASP)
250	125	8:1	0.363:1	83.2	1362	997.6
250	250	8:1	0.363:1	84.0	1467	929.6
250	500	8:1	0.363:1	85.1	1324	764.8
250	1000	8:1	0.363:1	84.6	1266	1011
250	2000	8:1	0.363:1	85.2	1385	943.2

**Table 5:** Experimental data of core displacement experiment.

Core Flooding Setup	Core Length/cm	Core Diameter/cm	Effective Porosity/%	Permeability /mD	Porosity/%	Oil Recovery/%
Simulated water	10	2.5	20.4	19.52	20.5	54.6
	10	2.5	20.6	19.69	21.6	55.2
Simulated Water + L^4^	10	2.5	20.9	19.89	21.3	60.3
	10	2.5	21.5	20.01	22.5	61.5

## Data Availability

Data are contained within the article.
